# Multi-campaign ship and aircraft observations of marine cloud condensation nuclei and droplet concentrations

**DOI:** 10.1038/s41597-023-02372-z

**Published:** 2023-07-20

**Authors:** Kevin J. Sanchez, David Painemal, Matthew D. Brown, Ewan C. Crosbie, Francesca Gallo, Johnathan W. Hair, Chris A. Hostetler, Carolyn E. Jordan, Claire E. Robinson, Amy Jo Scarino, Taylor J. Shingler, Michael A. Shook, Kenneth L. Thornhill, Elizabeth B. Wiggins, Edward L. Winstead, Luke D. Ziemba, Scott Chambers, Alastair Williams, Ruhi S Humphries, Melita D. Keywood, Jason P. Ward, Luke Cravigan, Ian M. McRobert, Connor Flynn, Gourihar R. Kulkarni, Lynn M. Russell, Gregory C. Roberts, Greg M. McFarquhar, Athanasios Nenes, Sarah F. Woods, Jeffery S. Reid, Jennifer Small-Griswold, Sarah Brooks, Simon Kirschler, Christianne Voigt, Jian Wang, David J. Delene, Patricia K. Quinn, Richard H. Moore

**Affiliations:** 1 NASA Langley Research Center, Hampton, VA 23681 USA; 2 Science Systems and Applications, Inc., Hampton, VA 23666 USA; 3 NASA Postdoctoral Program, Oak Ridge Associated Universities, Oak Ridge, TN 837830 USA; 4 National Institute of Aerospace, Hampton, VA 23666 USA; 5 Australian Nuclear Science and Technology Organisation, Lucas Heigths, NSW 2232 Australia; 6 Climate Science Centre, CSIRO Oceans and Atmosphere, Aspendale, Australia; 7 School of Earth and Atmospheric Sciences, Queensland University of Technology, Brisbane, Australia; 8 Engineering and Technology Program, CSIRO National Collections and Marine Infrastructure, Hobart, Australia; 9 School of Meteorology, University of Oklahoma, Norman, OK USA; 10 Atmospheric Sciences and Global Change Division, Pacific Northwest National Laboratory, Richland, USA; 11 Scripps Institution of Oceanography, La Jolla, CA USA; 12 Centre National de Recherches Météorologiques, UMR3589 Toulouse, France; 13 Cooperative Institute for Severe and High-Impact Weather Research and Operations, University of Oklahoma, Norman, Oklahoma USA; 14 Laboratory of atmospheric processes and their impacts (LAPI), ENAC/IIE, Ecole polytechnique fédérale de Lausanne (EPFL), Lausanne, Switzerland; 15 Institute for Chemical Engineering Sciences, Foundation for Research and Technology Hellas (ICE-HT/FORTH), Patra, Greece; 16 Stratton Park Engineering Company (SPEC), Boulder, CO 80301 USA; 17 Naval Research Laboratory, Monterey, CA USA; 18 University of Hawaii at Mānoa, Honolulu, HI USA; 19 Texas A&M University, College Station, TX USA; 20 Institute for Atmospheric Physics, DLR, German Aerospace Center, Oberpfaffenhofen, Germany; 21 Institute for Atmospheric Physics, University of Mainz, Mainz, Germany; 22 Center for Aerosol Science and Engineering, Washington University in St. Louis, St. Louis, MO USA; 23 University of North Dakota, Grand Forks, ND USA; 24 Pacific Marine Environmental Laboratory, NOAA, Seattle, WA USA

**Keywords:** Atmospheric chemistry, Climate change

## Abstract

*In-situ* marine cloud droplet number concentrations (CDNCs), cloud condensation nuclei (CCN), and CCN proxies, based on particle sizes and optical properties, are accumulated from seven field campaigns: ACTIVATE; NAAMES; CAMP^2^EX; ORACLES; SOCRATES; MARCUS; and CAPRICORN2. Each campaign involves aircraft measurements, ship-based measurements, or both. Measurements collected over the North and Central Atlantic, Indo-Pacific, and Southern Oceans, represent a range of clean to polluted conditions in various climate regimes. With the extensive range of environmental conditions sampled, this data collection is ideal for testing satellite remote detection methods of CDNC and CCN in marine environments. Remote measurement methods are vital to expanding the available data in these difficult-to-reach regions of the Earth and improving our understanding of aerosol-cloud interactions. The data collection includes particle composition and continental tracers to identify potential contributing CCN sources. Several of these campaigns include High Spectral Resolution Lidar (HSRL) and polarimetric imaging measurements and retrievals that will be the basis for the next generation of space-based remote sensors and, thus, can be utilized as satellite surrogates.

## Background & Summary

Atmospheric aerosol particles can act as cloud condensation nuclei (CCN) to nucleate water droplets forming clouds. Variability in the concentration of CCN modulates the optical properties of clouds, thereby significantly influencing the radiation budget^1–4^. The underlying physical and chemical properties that determine if a particle is CCN active under a set of given thermodynamic conditions are well known^5–7^. However, models struggle to accurately simulate real-world conditions due to their inability to simulate fine-scale processes and the complexity of particle sources, sinks, and evolution through numerous complex interactions^8–10^. The lack of available *in-situ* measurements, particularly over the oceans, prohibits significant progress in reducing the uncertainty in models and the overall impact of aerosol-cloud interactions on climate^11^. Clouds over the ocean are particularly sensitive to variations in CCN because there are lower concentrations of CCN particles relative to continental and polluted regions^12^. This sensitivity is known as the indirect effect, which states that a decrease in CCN particle concentration can cause a decrease in droplet concentrations^13^. To greatly expand our measurement quantity over the oceans, several remote detection methods have been published to estimate CCN concentrations via satellite^14–19^; however, these methods need evaluation to understand sources of uncertainty and systematic bias.

Since particle size plays a significant role in determining a particle’s ability to act as a CCN, some remote sensing methods rely on the particle size to optically derive the accumulation mode particles to obtain a CCN proxy^16,20,21^. Alternatively, particle extinction and optical depth, dominated by larger particles, are a common proxy for CCN concentrations^19,22–24^. The assumption based on particle size determining CCN activity of particles is based on the Kelvin effect, which describes the reduction in saturation vapor pressure due to the reduced curvature associated with larger particles^25–27^. The datasets presented here include both CCN measurements and metrics based on particle size distributions for comparisons with remote retrievals based on particle size.

Remotely retrieving aerosol properties in the presence of clouds is not currently possible. In cloudy regions, the gaps in CCN data can be filled with remote retrievals of cloud droplet number concentration (CDNC), representing the CCN concentration at the supersaturation level reached in the cloud^14,28,29^. Many of the campaigns included are aircraft based and include *in-situ* CDNC observations. Remote retrievals of CDNC via satellite have several necessary assumptions in the absence of vertically resolved data. Using these assumptions and readily available satellite cloud retrievals of cloud effective radius (r_e_), optical depth (τ), and temperature (T), CDNC can be derived (e.g., Seethala and Hováth^30^). The assumptions are that liquid water profiles are adiabatic and increase linearly with altitude. Also, CDNC is constant with altitude in-cloud and not influenced by entrainment, and the droplet spectral width is constant^31,32^. Rosenfeld *et al*.^14^ and Efraim *et al*.^33^ introduced methods to remotely acquire estimates of the in-cloud maximum supersaturation, after correcting for an assumed level of cloud droplet reduction due to inhomogeneous entrainment (complete evaporation of a fraction of the droplets). With the supersaturation and CDNC, comparisons between the retrieved CDNC and locally measured CCN spectra are possible.

Here we highlight the availability of *in-situ* CDNC, CCN measurements, and CCN proxies in marine conditions over a wide range of pristine and polluted conditions. Aircraft-based remote measurements (i.e., Light Detection, and Ranging and Research Scanning Polarimeter) complement these *in-situ* measurements. The remote measurements can act as “satellite proxies” as they are not currently on existing satellites. NASA plans to incorporate the HSRL and Polarimeter into the Atmosphere Observing System (AOS) satellite architecture^34–37^. Remote measurements of boundary layer particles are only possible in cloud-free paths, so utilizing both remote cloud-free particle measurements and remote CDNC is necessary to maximize the coverage of remotely estimated CCN concentrations. This extensive combination of datasets is ideal for comparing various remote detection methods with statistically significant calculations of skill. Furthermore, the variation of dataset location, anthropogenic pollution influence, season, and climate makes it possible to identify weaknesses in the various methods that may depend on the variations in atmospheric conditions and method assumptions.

## Methods

**Fig. 1:**
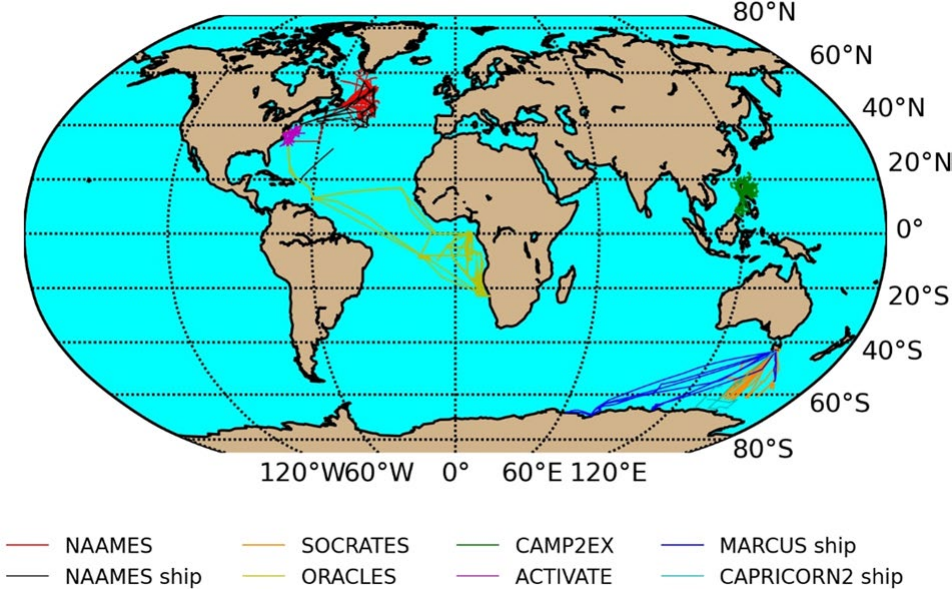
Ship and aircraft tracks for all included campaigns.

This section provides a general description of the measurement campaigns and instruments. Campaigns were chosen with a primary criterion of including marine boundary layer aerosol or cloud measurements and campaigns with diverse levels of continental and pollution sources. Figure 1. shows the location of the measurements, and Table 1 summarizes the local emissions and common cloud properties. Tables 2, 3 also contain summaries of the aircraft-based and ship-based instrument availability, respectively, for relevant *in-situ* measurements of CCN, CCN proxies, and CDNC for direct comparison with remote techniques. In addition, we have included measurements describing particle and cloud properties (size, composition, and pollution tracers) that are necessary to test method assumptions. Many campaigns are also prioritized because of the availability of High Spectral Resolution LIDAR (HSRL) and polarimeter measurements onboard the aircraft (Table 2), which can act as a surrogate for remote satellite measurements. While these particular HSRL and polarimeter capabilities are not currently available on satellites, they enable much more overlap with *in-situ* measurements than the rare overpasses of the available satellite-based CALIPSO lidar with *in-situ* measurements, and they may be in the future^36,37^.

**Table 1:** Campaign datasets used for the proposed study along with information on their locations, and typical aerosol, and cloud regimes.

Campaign	Location	Platform	Time of Year	Aerosol regimes	Main Cloud regime
ACTIVATE	North Atlantic	Aircraft	Jan.-Mar., May-June, Aug.-Sept.	Marine, Polluted	Shallow Cumulus, Postfrontal Stratocumulus
CAMP^2^EX	Western Tropical Pacific	Aircraft	Aug.-Oct.	Marine, Polluted, BB	Deep Cumulus
NAAMES	North Atlantic	Aircraft/Ship	Mar.-June, Aug.-Sept., Nov.	Marine, Polluted	Extra-tropical Shallow Cumulus, Postfrontal stratocumulus
ORACLES	South-East Atlantic	Aircraft	Aug.-Oct.	Marine, Polluted, BB	Stratocumulus
SOCRATES	Southern Ocean	Aircraft	Jan.-Feb.	Marine	Supercooled/Mixed Phase
CAPRICORN2	Southern Ocean	Ship	Jan.-Feb.	Marine	Supercooled/Mixed Phase
MARCUS	Southern Ocean	Ship	Oct.-Mar.	Marine	Supercooled/Mixed Phase

**Table 2:** Instruments used to measure relevant parameters for each airborne campaign and their approximate diameter range and temporal resolution. All airborne campaigns consist of measurements of aerosol size distributions, CCN, CDNC, HSRL and a Research Scanning Polarimeter (RSP), with the exceptions that SOCRATES and ORACLES 1–2 do not have RSP measurements.

Parameters	Instruments	Diameter range (µm)	Time resolution (s)	ORACLES (2016–2018)			NAAMES (2016–2018)	ACTIVATE (2020–2022)	CAMP^2^EX (2019)	SOCRATES (2018)
				2016	2017	2018				
Particle concentration and size distribution*	APS	0.5–20	1				x			
	LAS	0.09–7.5	1				x	x	x	
	UHSAS	0.06–1.0	1		x	x				x
	LDMA	0.01–0.55	~80	x	x	x				
	SMPS	0.01–0.3	~60				x			
	FIMS	0.01–0.6	1						x	
	CPC	>0.01, >0.003	1	x	x	x	x	x	x	x
	PCASP	0.10–3.1	1	x	x					x
Particle hygroscopicity	CCN counter	<1.0	1–300	x	x	x	x	x	x	x
	Nephelometer	<5.0	1	x		x	x	x	x	
	PSAP	<5.0	1	x		x	x	x	x	
Cloud droplet size distribution	CDP	2–50	1		x	x	x	x		x
	FCDP	1.5–50	1					x	x	
	FFSSP	1.5–50	1						x	
	CAS	0.51–50	1	x	x	x	x	x		
Drizzle or precipitation size distribution	HVPS	75–45000	1	x	x	x			x	
	2D-S	25–1280	1	x	x	x		x	x	x
	CIP	25–1550	1	x			x			
Water content	King	—	1	x	x	x				x
	Nevzorov probe	—	1						x	x
Pollutant and continental tracers	SP2	0.07–0.5^**^	1	x	x	x	x		x	
	AMS	0.04–1.0^***^	30	x	x	x	x	x	x	
	CO analyzer	—	1	x	x	x	x	x	x	
Remote cloud and particle measurements	RSP	—	~18	x			x	x	x	
	HSRL	—	10	x	x	x	x	x	x	x

^*^All aerosol particles are dried prior to measurement, except for PCASP measurements. Note, diameter measurements and limits are expressed as either aerodynamic, mobility or optical diameter.

^**^Instruments were set to various diameter ranges, but log-normal fits are used to account for black carbon mass outside the set range.

^***^The AMS transmission efficiency varies by particle size and slightly from instrument to instrument. Diameters are aerodynamic diameters.

**Table 3:** Instruments used to measure relevant parameters for each ship-based campaign and their approximate diameter range and temporal resolution. All campaigns consist of measurements of aerosol size distributions and CCN.

Parameter	Instruments	Diameter range (µm)	Time resolution (s)	NAAMES (2016–2019)	CAPRICORN2 (2018)	MARCUS (2017–2018)
Particle concentration and size distribution*	APS	0.5–20	1	x	x	
	nanoSMPS	0.005–0.335	300		x	
	SMPS	0.014–0.67	300		x	
	SEMS	0.01–1.0	300	x		
	CPC	>0.01, >0.003	1	x	x	x
	DMPS	0.002–0.8	300	x		
Particle hygroscopicity	CCN counter	<~1.0	1	x	x	x
Pollutant and continental tracers	SP2	0.07–0.5^**^	1	x		
	AMS	0.04–1.0^***^	300	x		
	ACSM	0.04–1.0^***^	300		x	
	CO analyzer	—	1			x
	Radon	—	1800	x	x	

^*^All aerosol particles are dried prior to measurement. Note, diameter measurements and limits are expressed as either aerodynamic, mobility or optical diameter.

^**^Instruments were set to various diameter ranges, but log-normal fits are used to account for black carbon mass outside the set range.

^***^The AMS and ACSM transmission efficiency varies by particle size and slightly from instrument to instrument. Diameters are aerodynamic diameters.

### North atlantic aerosols and marine ecosystems study (NAAMES)

NAAMES was conducted over four years through four deployment campaigns that included ship-based aerosol measurements on the *R/V Atlantis* in the North Atlantic^38^. Three of these deployments were complimented by C130 aircraft measurements of *in-situ* and remote aerosol and cloud measurements. The study focuses on improving the understanding of the ocean ecosystem-aerosol-cloud system of the western subarctic Atlantic through (1) characterizing plankton ecosystem properties during primary phases of the annual cycle and their dependence of environmental forcing, (2) determining how these phases interact to recreate each year the conditions for an annual plankton bloom, and (3) resolve how remote marine aerosols and boundary layer clouds are influenced by plankton ecosystems^38^. The four campaigns were conducted in November 2015, May-June 2016, August-September 2017, and March-April 2018. The March-April 2018 campaign is the only campaign not accompanied by aircraft measurements.

### Aerosol cloud meteorology interactions over the western atlantic experiment (ACTIVATE)

ACTIVATE took place off the coast of the United States and was based out of NASA Langley Research Center for over three years and six intensive aircraft campaigns to measure aerosol and cloud properties and robustly characterize aerosol-cloud-meteorology interactions. Instrumentation was deployed on two aircraft, the King Air and Falcon, for simultaneous remote and *in-situ* measurements by the two aircraft, respectively. The scientific goals of ACTIVATE are (1) to improve understanding and model representation of relationships between aerosol number concentrations, CCN, and cloud properties, (2) to generate a unique dataset for international model intercomparison and process-based studies, (3) to evaluate current remote sensing retrievals and prototypes for future satellite missions, and (4) develop improved satellite-based CDNC and CCN proxy retrievals^39^. The campaigns were conducted in February-March and August-September in 2020, January-March and May-June in 2021, and January-March and May-June in 2022.

### Observations of aerosol above clouds and their interactions (ORACLES)

ORACLES consisted of airborne (NASA P-3 and ER-2 Aircraft) *in-situ* and remote measurements over the south Atlantic, west of Africa^40^. Three field campaigns (2016–2018) were conducted during the southern African biomass burning season (August to October). The goals of the ORACLES study were (1) to determine the impact of African-produced biomass burning aerosol on cloud properties and the radiation balance over the south Atlantic and (2) to acquire process understanding of aerosol-cloud-radiation interactions and resulting cloud adjustments that can be applied to models.

### Clouds, aerosol, monsoon processes-philippines experiment (CAMP^2^Ex)

CAMP^2^Ex is an airborne mission conducted on the NASA P-3 and SPEC Lear-35 based in Clark, Philippines from 25 August-5 October 2019^41^. The campaign is designed to characterize the role of anthropogenic and natural aerosol properties in modulating the frequency and amount of warm and mixed-phase precipitation upstream of the North Subtropical Western Pacific’s Southwest Monsoon trough. Notable in CAMP^2^Ex was its wide variety of aerosol types, including a) biomass burning from Indonesian peatlands; b) the Metro Manila super plume; c) long range industrial pollution from the Maritime Continent through Southeast Asia and China, and finally pristine subtropical Pacific air masses. CAMP^2^Ex also cooperated significantly with the Office of Naval Research Propagation of Intraseasonal Oscillations (PISTON) mission, that provided the R/V *Sally Ride* with a host of lidars and radars^41^.

### Southern ocean clouds, radiation, aerosol transport experimental study (SOCRATES)

During SOCRATES, the NSF-NCAR GV aircraft sampled aerosol and clouds in and above the marine boundary layer along primarily north-south transects in January-February 2018, targeting areas of cyclones where models struggle to produce supercooled liquid water. In addition, the main goals of SOCRATES involved characterizing the structure of the marine boundary layer and free troposphere over the Southern Ocean, including the vertical and latitudinal distribution of aerosol and cloud properties^42–45^.

### Clouds aerosols precipitation radiation and atmospheric composition over the southerrn ocean (CAPRICORN2)

CAPRICORN2 consisted of ship-based measurements on the R/V *Investigator*. Measurements were conducted south of Tasmania, Australia (Fig. 1) and lead by the Australian Bureau of Meteorology. The objectives were to (1) characterize cloud, aerosol, and precipitation properties, boundary layer structure, biological production and cycling of dimethyl sulfide, atmospheric composition, and surface energy budget and latitudinal variations, (2) evaluate and improve satellite products (focusing on the NASA A-train and Global Precipitation Measurements mission cloud, precipitation, and surface heat flux products), and (3) evaluate and improve the representation of these properties in the Australian Community Climate and Earth-System Simulator model^42^. Measurements were conducted in January-February 2018, overlapping with the SOCRATES campaign.

### Measurements of aerosols, radiation and clouds over the southern ocean (MARCUS)

MARCUS is also a ship-based measurement study conducted south of Tasmania, Australia. Measurements were collected on a United States Department of Energy Atmospheric Radiation Measurement Program Mobile Facility deployed on the RSV *Aurora Australis* as it made four resupply trips to three Australian Antarctic bases (Mawson, Davis, and Casey) from October 2017 to March 2018. The objectives of MARCUS were to (1) understand synoptically varying vertical structure of Southern Ocean boundary layer clouds and aerosols, (2) quantify the sources and sinks of CCN and ice nuclei particles (INPs), including the role of local biogenic sources over spring, summer and fall, (3) quantify the mechanisms controlling supercooled liquid and mixed-phased clouds, and (4) advance retrievals of clouds, precipitation, and aerosol over the Southern Ocean^42^. The MARCUS campaign overlapped with both SOCRATES and CAPRICORN2.

### Instrumentation

Tables 2, 3 summarize the measurements made on each airborne and ship campaign, respectively. The available instrumentation listed in these tables is not exhaustive. This manuscript contains only CDNC, CCN, CCN proxies, and measurements necessary to identify particle physical and chemical properties and non-marine contributions to particle concentrations. Other measurements not discussed here are accessible through the original campaign archives referenced in the Data Records section. This section provides specific information for the listed instrument. All concentrations are reported with respect to standard temperature and pressure (273.15 K, 1013 hPa) unless indicated otherwise in the section below. Also, all particle measurements were collected during subsaturated conditions and further dried unless indicated otherwise. Tables 2, 3 identify which campaigns the instruments were used. Only in cases when there are different instrument models used for the same measurement, or if there are campaign specific details provided, will the campaign be discussed (Ex: Measurements collected from four different Condensation Particle Counters (CPC) models are discussed, so the text will indicate which model was used on which campaign). The uncertainty or relative statistical counting error of instruments counting particles is calculated as sqrt(N)/N, assuming Poisson statistics, where N is the measured number of particles. Measurements are made at 1 Hz frequency or averaged to provide 1 Hz frequency unless otherwise stated.

### Particle concentration and size distributions

During the NAAMES, ACTIVATE, and CAMP^2^EX airborne campaigns, Condensation Particle Counters models 3772, and 3025 (CPC, TSI Inc., St. Paul, MN) measures total condensation nuclei (CN) concentration with lower cut-off diameters of 0.01 µm, and 0.003 µm, respectively. The model 3772 CPC is also used on the CAPRICORN2 and MARCUS campaigns. During the ORACLES airborne and NAAMES ship campaigns, a model 3010 CPC (TSI Inc., St. Paul, MN) measures total submicron particle concentration and has a lower cut-off diameter of 0.01 µm. For the SOCRATES campaign, a model 3760 A CPC (TSI Inc., St. Paul, MN) is used, with a lower cut-off diameter of 0.01 µm. The Laser Aerosol Spectrometer (LAS Model 3340, TSI Inc., St. Paul, MN) measures particle optical diameter distributions between 0.1 µm and 3.5 µm. The Aerodynamic Particle Sizer (APS Model 3321, TSI Inc., St. Paul, MN) characterizes the aerodynamic diameter of large dry particles, including dust, and has a size range of 0.5–20 µm; however, the inlet cut size is approximately 5 microns^46^. On CAPRICORN2, the APS Model 3320 (TSI Inc., St. Paul, MN) is used. The APS has 50 diameter bins. The long differential mobility analyzer (LDMA Model 3934, TSI Inc., St. Paul, MN) measures aerosol size distributions in the 0.01–0.55 µm diameter range. During ORACLES in 2016, a leak affected the LDMA, which is accounted for with an altitude-dependent correction. Also, the 2016 and part of the 2017 datasets have been multiplied by a factor of 1.6 to account for particle loss in a partially blocked 0.7 µm impactor upstream of the LDMA. The LDMA operates with a lag chamber that stores the air sample during a 60 second voltage scan. The chamber is then flushed for 20 seconds with ambient air. The aircraft Scanning Mobility Particle Sizer (SMPS) is a custom-built system using a Differential Mobility Analyzer (DMA) to measure the particle size distribution over a scan time of ~60 seconds. The SMPS scanned from 0.01–0.30 µm mobility diameter using a long DMA (Model 3081, TSI Inc., St. Pail, MN). For the SMPS and LDMA scanning sampling techniques, caution should be used when examining measurements collected during and near ascents and descents. The Fast Integrated Mobility Spectrometer^47^ detects particles in the size range of 0.01–0.60 µm simultaneously, allowing a 1 Hz time resolution. The Ultra-High-Sensitivity Aerosol Spectrometer (UHSAS, DMT, Boulder, CO) measures particle size distribution between 0.060 and 1.00 µm diameter. During ORACLES, there are two UHSAS instruments. On CAPRICORN2, a long DMA SMPS (Model 3080 + 3081, TSI Inc., St. Paul, MN) is used with a model 3772 TSI CPC to measure particle mobility diameters from 0.015 – 0.670 µm mobility diameter over 5-minute scans and a nano SMPS (Model 5.420, GRIMM, Ainring, Germany), with an M-DMA installed, measures particle mobility diameter from 0.005–0.350 µm over 5-minute scans. The Scanning Electrical Mobility Sizer (SEMS, model 138, 2002, BMI, Hayward, CA) measures dry particle size distributions from 0.01–0.90 μm diameter with 5-minute scans. A differential mobility particle sizer (DMPS, University of Vienna)^48^ measures the number size distribution from 0.02–0.80 μm diameter with 5-minute scans. Finally, the Passive Cavity Aerosol Spectrometer Probe (PCASP, DMT, Boulder, CO) measures particles optically from 0.1 to 3.0 µm at ambient relative humidity.

### Particle and gas composition

Remote particle composition measurements are limited and highly uncertain. Particle composition is necessary to include when validating remote CCN measurements since particle composition affects its ability to act as CCN, and therefore is likely a source of uncertainty. Furthermore, errors in remote measurements of marine CCN concentrations may vary with continental and pollution influences, so categorizing measurements based on the particle sources is essential to identify sources of uncertainty.

Submicron particle composition is analyzed with a high-resolution time-of-flight aerosol mass spectrometer (AMS, Aerodyne Research Inc., Billerica, MA)^49^ that typically measures bulk non-refractory inorganic (sulfate, ammonium, nitrate, chloride) and organic components. Some AMS modes can measure single-particle composition, but these measurements are not widely available for airborne campaigns. The approximate size range of measured particles is 0.06–0.60 µm vacuum aerodynamic diameter and can vary slightly by instrument. No correction has been applied to account for mass outside this diameter range. The uncertainty is about 50% and 25% for airborne and ship measurements, respectively, based on sample scan times, processing assumptions, and instrument limitations. AMS ship-borne measurements typically have longer sample scan times than airborne measurements due to the relatively slow change in air mass due to the slow progress of the ship compared to aircraft. The longer sampling time for ship measurements reduces the uncertainty relative to the aircraft measurements^50^. Although the AMS collection efficiency varies by instrument and with atmospheric conditions and is sometimes not corrected for or assumed in the published datasets, the non-refractory particle composition ratio is still a relevant and valuable quantity for evaluating bulk particle chemical properties. On aircraft, during NAAMES, ACTIVATE, and CAMP^2^EX, the AMS measures particle mass concentrations in V-mode (high sensitivity) at 30 second intervals.

During the NAAMES campaigns, the AMS on the *R/V Atlantis* had much longer sample cycle times. Each sample cycle lasts a total of 5 minutes. During each cycle, the ambient aerosol is sampled in V-mode (high sensitivity) for 2 min, W-mode (high resolution) for 1 min, and event-trigger-mode (single particle composition) for 2 min. Supermicron particles were removed prior to sampling with a sharp-cut cyclone (SCC 2.229, BGI Inc. US).

Finally, during the CAPRICORN2 campaign on the *R/V Investigator*, an Aerosol Chemical Speciation Monitor (ACSM) measured particle mass compositions similar to the AMS. The ACSM measures the same components as the AMS plus methanesulfonic acid (MSA). The upper size cut is approximately 1.0 µm diameter, and the transmission efficiency drops below 0.1 µm in aerodynamic diameter.

### Pollutants

Refractory black carbon particle mass is measured with a Single Particle Soot Photometer (SP2, DMT, Boulder, CO) to identify anthropogenic pollution. The SP2 derives black carbon mass by measuring laser-induced particle incandescence for particles between 0.07 and 0.50 µm in diameter, assuming a black carbon density of 1.8 g cm^−3^. The SP2 sampling rate on the NAAMES ship and all aircraft campaigns are 60 s and 10 s, respectively. The SP2 instruments used in these studies are calibrated to similar but slightly different diameter ranges, reported in the originally published campaign datafiles. Black carbon mass outside the calibrated range is still accounted for using a log-normal fit to the distributions. For SP2 measurements collected in-flight, where sampling rates are increased due to the considerable distance covered by the aircraft, the log-normal fit is applied to the flight-averaged data to produce a correction coefficient that is applied to the entire flight. The correction coefficient typically increases the black carbon mass by less than 10%. During the NAAMES and ORACLES aircraft campaigns and MARCUS ship campaigns, the carbon monoxide mixing ratio is measured with a CO/CO_2_ gas analyzer (Los Gatos Research, San Jose, CA). During the ACTIVATE and CAMP^2^EX campaigns, the carbon monoxide mixing ratio is measured using a G2401-m in-flight Gas Concentration Analyzer (Picarro Inc., Santa Clara, CA). On both the NAAMES and CAPRICORN II ship campaigns, radon activity concentration was measured in mBq m^−3^ as a tracer for continental influences on marine air masses. Radon is a naturally occurring radioactive gas emitted by soil and rocks and has a half-life of about 3.8 days. In both campaigns, a dual-flow-loop, two-filter detector^51^ is used for measurements.

### In-cloud measurements

**Fig. 2:**
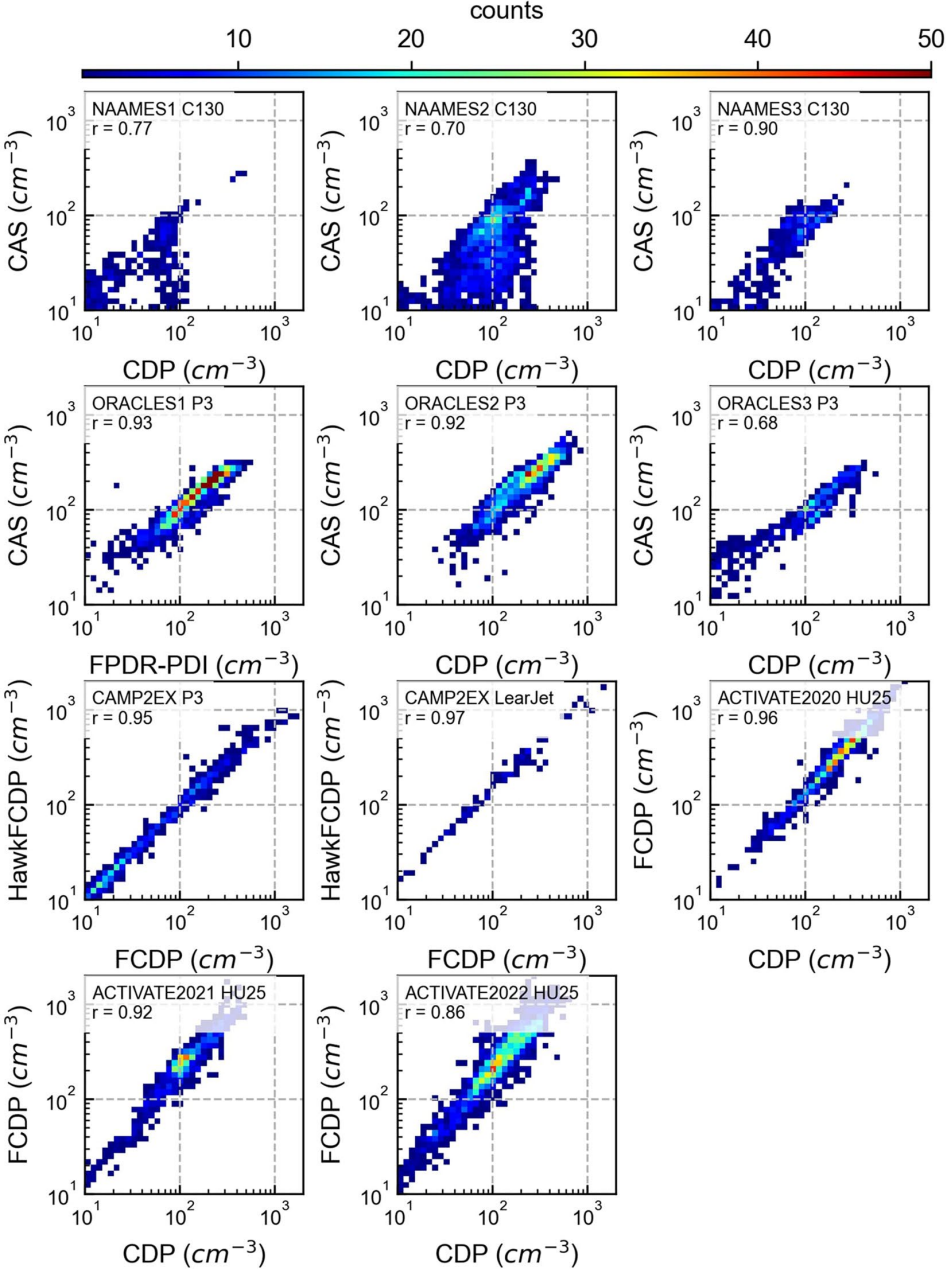
Comparisons of two *in-situ* CDNC measurements. The measurements are collected by two of the following instruments: CDP, CAS, FCDP, and HawkFCDP. *In-situ* measurements are averaged to 10-second intervals and exclude cloud edges with a 10 second buffer. Grid values represent the total number of counts at the observed values for the campaign.

The Cloud Droplet Probe (CDP, DMT, Boulder, CO) measures cloud droplet size distributions for droplets ranging from 2–50 µm in diameter. The Fast Cloud Droplet Probe (FCDP, SPEC inc. Boulder, CO) also measures cloud droplet distributions of droplets from 1.5–50 µm in diameter. Both the CDP and FCDP are sensitive to some coarse mode aerosol. The Fast Forward Scattering Spectrometer Probe (FFSSP, SPEC inc., Boulder, CO) also measures particles from 1.5 to 50 µm in diameter. The Cloud-Aerosol Spectrometer (CAS, DMT, Boulder, CO) measures particles ranging from 0.51–50 µm in diameter at ambient relative humidity. The CDP, FCDP, FFSSP, and CAS measure both liquid and ice particles and cannot distinguish between the two phases. The Cloud Imaging Probe (CIP, DMT, Boulder, CO) measures the size of particles from 25–1550 µm with 25 µm bins. The Two-Dimensional Stereo Optical Array Probe (2D-S, SPEC inc., Boulder, CO) images cloud particles to obtain droplet sizes ranging from 25–1280 µm diameter range. For both the 2D-S and the CIP, the smallest bins (<50 µm) are excluded from the aggregated datasets due to the large uncertainty associated with them. The High-Volume Precipitation Spectrometer (HVPS, SPEC inc., Boulder, CO) combines the 2D-S optoelectronics with optics and probe tips designed to minimize shattering and image particles as large as 1.92 cm with a 150 µm pixel resolution. The CIP, HVPS, and CPI can identify the particle phase. There is much uncertainty in the particle size and concentration from optical probes that are introduced in the processing of the data due to the lack of consensus on how to handle partially imaged particles, shattered particles, out-of-focus particles, and other caveats that require assumptions. In addition, smaller particles (<150 µm) are particularly uncertain in size because they cover only a small number of pixels and have a highly uncertain depth of field. The Hawkeye is a combination of four probes in one and was originally developed by SPEC Inc. to fly on the NASA Global Hawk unmanned aerial vehicle. The four probes include an FCDP, two 2D-S probes, one of which is modified to have a 50 µm resolution and size range from 50–6400 µm, and finally a Cloud Particle Imager (CPI, SPEC inc., Boulder, CO) which images particles with 2.3 µm bin resolution and has a size range of 2.3–2300 µm. Measurements from the Hawkeye FCDP are referred to HawkFCDP in Fig. 2. The King probe (DMT, Boulder, CO) measures the cloud liquid water content (LWC) from hot-wire measurements with an uncertainty of 15%^52^. The Nevzorov probe^53^ has two separate sensors, one for measuring cloud LWC and one for measuring cloud total water content (TWC, i.e., LWC + ice water content) with an uncertainty of 20%. Vertical velocity is derived with differential pressure measurements, is corrected for aircraft heading and has an uncertainty of ±0.1 m s^−1^.

Some probe data is excluded from this data descriptor after careful analysis. The Flight Probe Dual Range Phase Doppler Interferometer (FPDR-PDI, Artium Technologies Inc., Sunnyvale, CA) was only used during the ORACLES campaign and is a redundant measurement as it overlaps entirely with the CDP and CAS size range. For this overlapping size range, Gupta *et al*.^54^ determined that the CAS and CDP were the better measurements for 2016 and 2017–2018, respectively; therefore, the FPDR-PDI measurements are excluded. In addition, during SOCRATES, the Precipitation Imaging Probe (PIP, DMT, Boulder, CO) and a 2-Dimensional Cloud probe (2D-C) which measure large precipitation-sized particles, were both deemed unusable due to a problem with the time record and degraded image quality, respectively^55^.

### Remote measurements

The NCAR nadir/zenith-pointing High-Spectral Resolution Lidar (HSRL) measured the vertical curtain of aerosol extinction, backscatter, and depolarization at a 0.532 µm wavelength^56^. Similarly, the NASA nadir-pointing HSRL made the same vertical curtain measurements and additionally measured backscatter and depolarization at 1.064 µm wavelength^57^. With these measurements, particle type^58,59^ and CCN concentration can be estimated based on methods developed by Georgoulias *et al*.^17^, who used CALIPSO lidar measurements to estimate surface CCN concentration. Additionally, traditional remote vertically integrated aerosol optical depth (AOD) and extinction measurements are directly comparable to *in-situ* CCN measurements as a baseline comparison. The HSRL can be considered a useful surrogate of what could be obtainable from future satellites^36,37^. Also, the HSRL has a higher signal-to-noise ratio and more overlap with *in-situ* measurements than the CALIPSO polar-orbiting satellite, enabling a better statistical comparison.

Similarly, many campaigns utilized a Research Scanning Polarimeter (RSP), which can extract aerosol products by measuring the upwelling total and polarized reflectance at multiple angles and spectral bands using refractive telescopes. With these measurements, the microphysical aerosol properties from polarimetry (MAPP) algorithm derives a bi-modal particle size distribution ranging from 0.094 to 5.1 µm^35^. RSP measurements are not currently available on satellite measurements.

### Particle hygroscopic growth and CCN

**Fig. 3:**
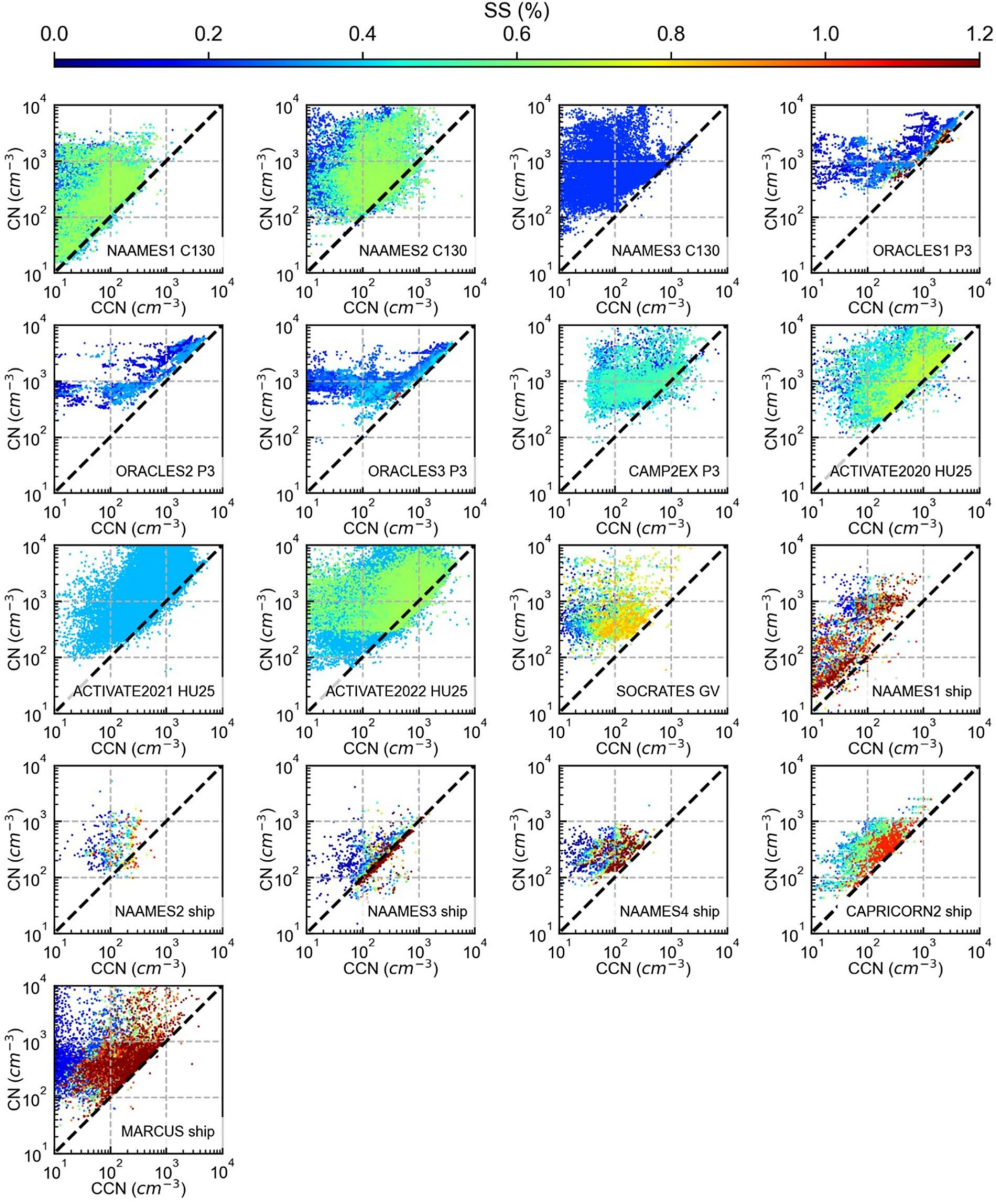
Comparisons of *in-situ* CCN measurements, at various supersaturation levels, with total CN for both aircraft and ship-based campaigns. Aircraft measurements are 10-second averages, ship measurements are 5-minute averages, except for CAPRICORN2, which are hourly averages.

**Fig. 4:**
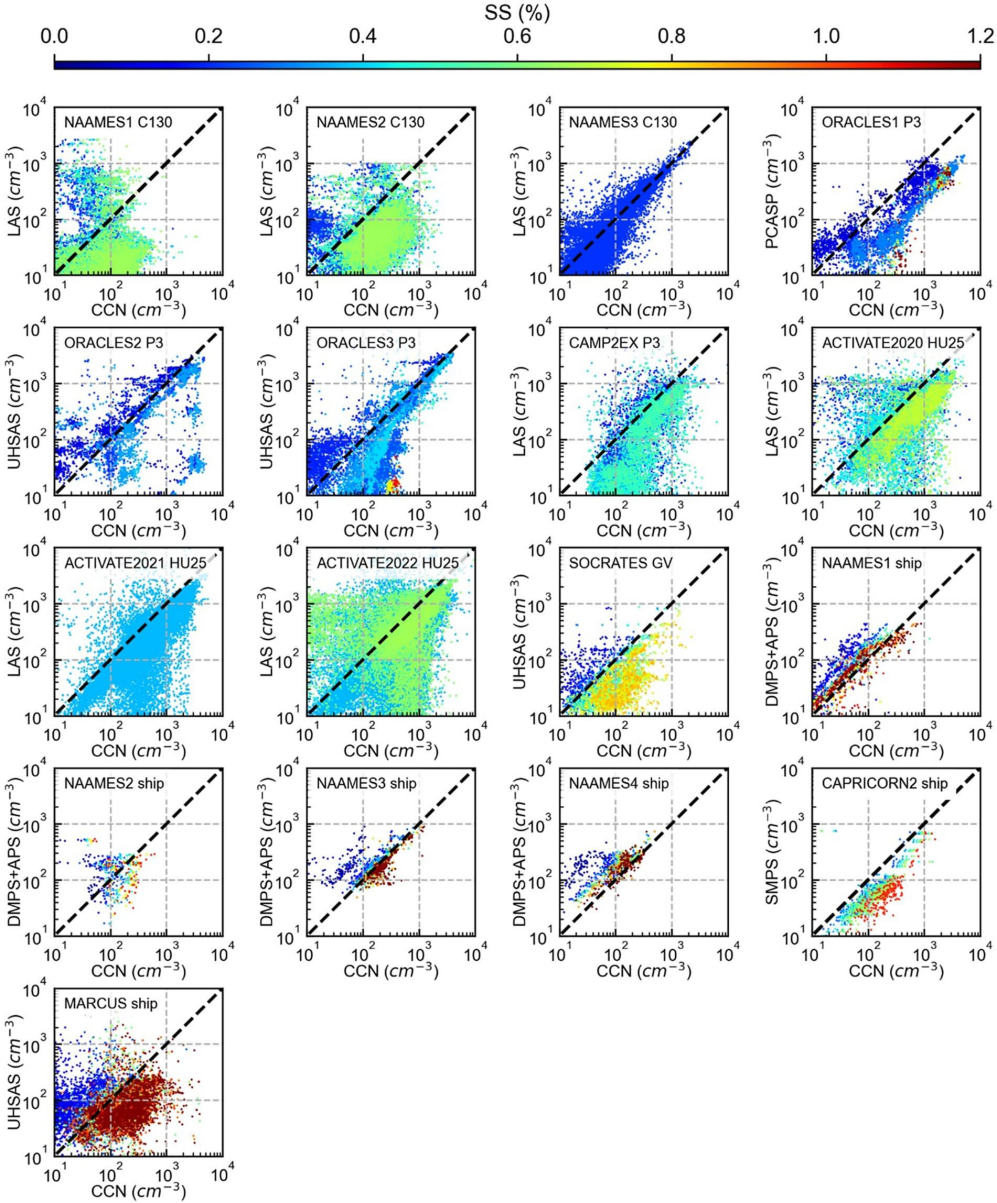
Comparisons of *in-situ* CCN measurements at various supersaturation levels and total CN greater than 100 nm in diameter for both aircraft and ship-based campaigns. CN greater than 100 nm in diameter is derived from several instrumentation, depending on what is available in each campaign. All size distributions measurements are of dried particles except for the PCASP measurements shown for ORACLES1 because no dry particle distribution was available. Aircraft and ship measurements shown are 10-second and 5-minute averages, respectively, except for CAPRICORN2, which are hourly averages.

Two 3-wavelength integrating nephelometers (0.450, 0.550, 0.700 µm) (Model 3563, TSI, St. Paul, MN) measure the total scattering, one at dry RH (<40%) and the other at a high RH (~80%) with a 25% uncertainty. Scattering coefficients are corrected for truncation errors using Anderson and Ogren^60^. Similarly, the aerosol absorption is derived from two Radiance Research 3-wavelength (0.470, 0.532, 0.660 µm) Particle Soot Absorption Photometers (PSAP), one at dry RH (<40%) and the other at high RH (~80%) with sometimes up to 50% uncertainty in coarse aerosol (rare in the marine boundary layer relative to the continental boundary layer). Data were corrected for a variety of errors using Virkkula^61^. The Nephelometer and PSAP pairs can be used to calculate the aerosol extinction coefficient by first using the measured angstrom exponent to convert the scattering coefficient to 0.532 µm then taking the sum of the absorption and scattering. With scattering coefficients for wet and dry air, the aerosol extinction at 0.532 µm is derived for the measured ambient humidity by assuming a single-parameter monotonic growth curve^62,63^. These measurements are particularly useful for testing assumptions involving hygroscopic growth corrections for remote measurements made at ambient relative humidity. During some of the ACTIVATE flights, the drying of the dry nephelometer was suboptimal, resulting in a smaller ambient relative humidity range for particle growth correction. Only measurements from campaigns with both wet and dry scattering measurements are helpful for this analysis as both are necessary to derive the hygroscopic influence on particle scattering at ambient relative humidity. The theoretical cut size for the nephelometers and PSAPs is 3–4 µm^46,64^. The Stream-wise thermal gradient continuous-flow CCN counters (DMT, Boulder, CO) and custom-built miniature versions^65^ measure CCN spectra over a scanned range of supersaturations and CCN concentrations at constant supersaturations. The constant and scan supersaturation ranges vary by campaign (Figs. 3, 4).

## Data Records

An aggregated dataset, consisting of time series with all *in-situ* aircraft or ship campaign measurements presented in Tables 2, 3, is available through Dryad^66^. All missing or invalid data flags are converted to ‘Na’. Some datasets have already been filtered for inlet shattering in-cloud and measurement contamination from ship exhausts; however, methods of filtering ship exhaust vary by campaign. For the NAAMES ship campaigns, the research ship exhaust was identified and filtered out based on the wind direction relative to the ship exhaust and total particle counts. For CAPRICORN2, wind direction, total particle counts, black carbon particle concentration, and CO and CO_2_ measurements were also utilized in filtering ship exhaust^67^. Finally, the MARCUS ship exhaust contamination periods are identified and filtered using total particle counts and CO measurements^67^. The aggregated dataset is further filtered to eliminate measurements influenced by in-cloud inlet shattering and averaged at 10-second intervals for aircraft measurements and 5-minute intervals for ship measurements (except for CAPRICOR2, which is only publicly available at hourly averaged intervals). While the remote HSRL and RSP measurements are briefly discussed, they are not included in the aggregated dataset. Remote retrievals methods of aerosol, CCN, and CCN proxies are constantly being updated and improved. Therefore, the raw data may be necessary to validate methods developed in the future. The remote measurement can be found in each campaign’s data archive, discussed in the last paragraph of this section. Evaluating current published remote retrieval methods is a focus of ongoing work.

In the discussed campaigns, many instruments measure particle, cloud, drizzle, and precipitation size distributions (discussed in the methods section and shown in Tables 2, 3). Almost all have different size range limitations. Here we create value-added products and size distinctions in the aggregated dataset to highlight two other variables related to CCN concentration and to create consistency between instrumentation products with varying limitations. First, we create a CCN proxy based on particle size from instruments that measure submicron particles. The proxy is calculated as the total number of particles > 0.1 µm diameter. How well this proxy represents the CCN concentration depends on the particle composition and supersaturation (Fig. 4). In the aggregated dataset, this CCN proxy is identified by variable names that start with the instrument abbreviation and end with “ > 0.1” to denote that they represent only the particles greater than 0.1 µm diameter (e.g., LAS_ > 0.1). The general quantity is referred to as CN_>0.1_. Note that this proxy’s upper particle size limit is instrument-dependent and shown in Tables 2, 3 (typically >0.6 µm). CN_>0.1_ is sometimes a poor proxy, particularly in low supersaturations conditions and when the particles are hydrophobic^68^, but it takes advantage of the fact that a particle’s size greatly influences its ability to thermodynamically activate and form a cloud droplet due to the Kelvin effect^21,25–27^ and has the advantage of often being consistently available, unlike CCN concentrations. Notably, the chemical composition significantly impacts CCN activation ability at smaller sizes and higher supersaturations^65^. Second, the in-cloud measurements of cloud droplet number concentration, total water content, and diameter, excluding drizzle and precipitation, are designated to measurements of particles that are <50 µm diameter and have a minimum size of 1.5–3 µm in diameter (instrument dependent). In the literature, several thresholds are proposed to define at what size coalescence is efficient, leading to drizzle and precipitation. Such thresholds typically range from 40 to 80 µm diameter^69^. In addition, a typical upper diameter limit for instruments measuring the smallest cloud droplets is 50 µm. This has caused the sub 50 µm diameter droplet concentration to often be reported as the non-drizzling cloud-droplet number concentration. For these reasons, we integrate cloud droplet properties for droplets up to 50 µm diameter. These cloud droplet measurements are identified by variable names that end with “<50” to denote that these variables represent cloud droplet measurements of droplets less than 50 µm diameter. Measurements of droplets greater than 50 µm in diameter are separately defined with variable names ending with “>50”. These likely represent drizzle or precipitation-sized droplets and could help identify the impact of drizzle and precipitation on remote sensing retrievals estimating cloud properties. Specifically, the CIP and 2D-S are available in the aggregated dataset as total droplet concentrations greater than 50 µm diameter.

If full-size distributions or different thresholds instead of the integrated value-added products, the data can be obtained from permanent archives. Measurements are retrieved from several data repositories to create the aggregated dataset. The original data for the NASA-led NAAMES^70^, ACTIVATE^71^, ORACLES^72^, and CAMP^2^EX^73^ are available through the Earthdata Atmospheric Science Data Center Sub-Orbital Order Tool (SOOT) (https://asdc.larc.nasa.gov/soot/). SOOT will soon incorporate the ability to download multiple variables in a custom merge file. The CAPRICORN2 CPC, CCN, and ACSM measurements are available through the Commonwealth Scientific and Industrial Research Organization Data Access Portal^74^. The remaining CAPRICORN2 data in the aggregated dataset have not been previously published. The measurements are archived under several single digital object identifiers (DOIs) for the SOCRATES and MARCUS campaigns. All the SOCRATES data is available through the NCAR/UCAR Earth Observatory Laboratory Data Archive, where the HSRL^75^, 2D-S^76^, CCN^77^ and remaining data^78^ are available via separate DOIs. Finally, the MARCUS measurements are available through the Atmospheric Radiation Measurements Data Center. The ship navigation^79^, UHSAS^80^, CPC^81,82^, CCN^82,83^, CO^84^, and meteorological^85^ data are available through individual DOIs. SOOT will soon incorporate the ability to download multiple variables in a custom merge file.

## Technical Validation

### Cloud droplet measurements

The *in-situ* CDNC measurements from the campaigns have redundant instrumentation for validation. Figure 2 shows CDNC comparisons from two separate instruments for all aircraft-based campaigns except for SOCRATES. SOCRATES is the only campaign without a redundant CDNC measurement; however, a King-probe LWC measurement is available and compared to the integrated CDP CDNC volume (r = 0.90). Before comparing measurements from the two instruments, LWC measurements were used to determine if the measurements were in or out of the cloud. All instruments that measured LWC were time synced by maximizing the cross-correlation between measurements and visually verified. Following the time sync, measurements were determined to be in-cloud when any instrument’s cloud LWC was greater than 0.02 g m^−3^. Then a 10-second buffer is applied to remove cases at cloud edges (i.e., measurements within 10 seconds of exiting or entering a cloud are excluded). Finally, a 10-second average is applied to the CDNC measurements to produce Fig. 2.

The CDNC comparisons generally show good agreements (r ≥ 0.70). The worst correlations were from the NAAMES airborne campaigns in comparisons with a CDP and a CAS because there were often problems with the CAS significantly undercounting the CDNC. For this reason, it is recommended that the CDP measurements are prioritized over the CAS for CDNC for the NAAMES dataset. Excluding the CDNC comparisons from NAAMES, the correlations are strong (r ≥ 0.89). It is worth noting the FCDP may provide more accurate measurements than the CDP, CAS, or FFSSP in conditions where CDNC is high (>200 cm^−3^) due to the higher possibility of coincidence in the CDP, which leads to undercounting and over-sizing of cloud droplets^86–88^.

### Aerosol and CCN measurements

The *in-situ* CCN concentration is compared to total CN (Fig. 3) and CN greater than 0.1 µm (CN_>0.1_) (Fig. 4) for all aircraft and ship-based campaigns. For aircraft-based subsaturated measurements, TWC is used to exclude in-cloud measurements. For out-of-cloud measurements, the TWC is required to equal 0 g m^−3^, a 10-second buffer is applied to remove measurements near the cloud that may be influenced by inlet shattering (i.e., measurements within 10 seconds of exiting or entering a cloud are excluded), and a 10-second average is applied. Ship-based measurements are averaged at 5-minute intervals, except for the CAPRICORN2 dataset, which is provided at hourly averaged intervals.

As expected, the comparison in Fig. 3 shows CCN concentration is less than or equal to the CN concentration (within the measurement error), with measurements at higher supersaturations matching more closely with the CN concentration than at lower supersaturations. As discussed in the introduction, remote detection methods often approximate CCN concentration by differentiating particles by size. Therefore, CN_>0.1_ (a value-added product defined in the Data Records section as particles greater than 0.1 µm diameter) acts as a relatively good proxy for CCN concentrations^21^. Comparing CCN and CN_>0.1_ is not a one-to-one comparison because particles greater than and less than 0.1 µm diameter both may or may not be CCN active depending on the level of supersaturation and particle composition. However, particles above 0.1 µm diameter are more likely to act as CCN than smaller ones, causing some correlation between the two variables, and are a necessary approximation in the absence of CCN measurements.

## Usage Notes

The measurements shown in this data descriptor and other *in-situ* measurements in Tables 2, 3 are processed from the publicly available data repositories using a customized python code. The python code is available for transparency; however, due to the large number of data files needed for input and the need to account for variable file formats, the output files with the time-synced datasets are available in ‘.csv’ format.

This aggregated dataset is compiled with the purpose of validating remote retrievals of CDNC, CCN, and CCN proxies from satellite, HSRL, and RSP data available at the time of the campaigns. The goal is to improve satellite retrievals and model representation of aerosol and cloud properties. Other possible uses of this dataset include:

- Statistical studies quantifying the influence of regional pollutant perturbations and environmental differences on marine particle and cloud properties. Particularly precipitation susceptibility and cloud lifetime.
- Evaluate model simulated aerosol and cloud properties. Specifically, the model representation of particle source, transport, composition, and CCN activity when forming clouds.
- Aerosol and cloud microphysical studies with CCN and CDNC closure studies to identify the sensitivity of cloud microphysical and radiative properties to the aerosol and boundary layer dynamics.
- Validating CDNC, CCN, and CCN proxy detection methods via remote Satellite, HSRL, and RSP methods that are developed in the future, assuming the raw measurements can be reprocessed based on the new methods.

## Data Availability

The python code is available for transparency and use through Dryad (Data Citation 1^66^); however, due to the large number of data files needed for input and the need to account for variable file formats there are many custom aspects to the code that are included for specific datasets making the code complicated. The coding environment dependencies and versions are included in a .yml file.
